# Immunomodulatory Changes Following Isolated RF Ablation in Colorectal Liver Metastases: A Case Report

**DOI:** 10.3390/medicines6020056

**Published:** 2019-05-13

**Authors:** Nona Janikashvili, Kumar Jayant, Nino Kikodze, Ketevan Mazmishvili, Ia Pantsulaia, Bynvant Sandhu, Mauro Podda, Manana Iobadze, Tamta Azrumelashvili, Malkhaz Mizandari, Nagy Habib, Tinatin Chikovani

**Affiliations:** 1 Department of Immunology, Tbilisi State Medical University, 0186 Tbilisi, Georgia; nonajanikashvili@gmail.com (N.J.); nino_kikodze@yahoo.com (N.K.); ianat73@mail.ru (I.P.); tamta_azrumelashvili@yahoo.com (T.A.); tinchikovani@gmail.com (T.C.); 2 Department of Surgery and Cancer, Imperial College London, London W120HS, UK; b.sandhu@imperial.ac.uk (B.S.); nagy.habib@imperial.ac.uk (N.H.);; 3 General, Emergency and Robotic Surgery Unit, San Francesco Hospital, 08100 Nuoro, Italy; mauropodda@ymail.com; 4 Department of Interventional Radiology, Tbilisi State Medical University, 0144 Tbilisi, Georgia; mananaiobadze@hotmail.com (M.I.); mgmizandari@gmail.com (M.M.)

**Keywords:** colorectal cancer, immunomodulation

## Abstract

**Background:** Colorectal cancer (CRC) is the third most common cancer worldwide and the second leading cause of cancer-related deaths in developed countries. The liver is the most prevalent site of metastasis from CRC. Currently, the gold-standard treatment for colorectal liver metastases (CLMs) is surgical resection. However, depending on the pattern of the disease, a significant number of patients may require different approaches alone or in combination with surgery, including thermal ablation (radiofrequency (RFA) or microwave (MWA) ablation) or transarterial liver-directed therapies, although the latter is not yet part of the standard treatment for CRC liver metastases. **Methods and Results:** We present the case of a 63-yearold man with bilobar CLM who was treated with transarterial embolization (TAE) and RFA followed by chemotherapy. A post-RFA study of immune parameters revealed the downregulation of CD39 expression in the circulating CD4^+^ T cell population and a reduction of the serum levels of cytokines IL-10, TGF-β, IFN-gamma and IL-17, which positively correlated with the diminished serum level of gamma-glutamyl transferase (GGT) and the subdued inflammatory markers: the neutrophil/lymphocyte ratio (NLR) and platelet/lymphocyte ratio (PLR). Later, the patient underwent chemotherapy. Liver failure developed within two years and nine months following tumour ablation, leading to the death of the patient. **Conclusions:** However, the denial of adjuvant chemotherapy by the patient gave us the opportunity to assess the immunomodulatory changes following RFA in the absence of any other therapeutic modalities.

## 1. Introduction

Colorectal cancer (CRC) is the fourth most common cancer worldwide and the second most common cause of cancer-related deaths in Western countries [1,2]. The National Cancer Institute Surveillance, Epidemiology and End Results (SEER) Database estimated 140,250 new cases of CRC and 50,630 deaths in 2018 contrary to other common cancers including lung, breast and prostate with a median age of diagnosis of 67 years [3]. The liver is the most frequent site of the metastatic spread of CRC, and about 25%–30% of patients with CRC develop liver metastases during their lifetime. Almost 20% of CRC patients present with metastasis at first consultation, and the five-year survival rate for untreated liver metastases is less than 3% [4].

Liver resection is the treatment of choice for colorectal liver metastases (CLMs) with a five-year survival rate reaching up to almost 60% in resections performed with curative intent. However, only 20% of patients are eligible for liver resection [5,6]. The treatment of CLM requires a multidisciplinary approach involving surgeons, interventional radiologists, and oncologists. Different treatment modalities can be offered alone or in combination to achieve a cure or to prolong the overall survival of patients [7,8].

Radiofrequency ablation (RFA) is a technique that causes tumour destruction by the induction of hyperthermia [9,10]. RFA can be used to directly treat liver tumours or to assist in liver resection. Either alone or in combination with other treatment modalities, RFA has been shown to be a safe and feasible approach in patients with unresectable hepatic tumours, especially in situations where liver resection or transplantation is not possible owing to impaired liver function and underlying co-morbid conditions [11,12]. 

The RF irradiation initiates ionic agitation and generates high-temperature focal hyperthermia (150 °C) in tumour tissue and induces irreparable cellular damage through coagulative necrosis. In addition to tumour ablation, various preclinical and clinical studies have ascribed the potential of RF in fostering an antitumour immune response by virtue of its immunomodulatory effects. The debris produced following RF-induced coagulative necrosis instigates tumour antigens and chemokines, which entices immunoprotective infiltrates, macrophages, neutrophils, DCs, and NK cells. DCs activate the nuclear factor kappa-light-chain-enhancer of activated B cell (NF-κβ) pathways, which stimulate CD8^+^ and CD4^+^ T lymphocytes and promote a systemic immune response [13,14,15].

Moreover, other modalities such as transarterial chemoembolization (TACE) and transarterial embolization (TAE) have been used increasingly in the treatment of unresectable liver tumours owing to the precisely targeted, minimally invasive, repeatable, and well-tolerated approach of these methods, which not only act as adjuncts to RFA but also enhance their efficacy [16,17]. In addition, combining these modalities with RFA helps in achieving maximum tumour ablation while preserving maximum liver parenchyma function [18,19]. 

Various studies have supported improved five and ten-year survival rates following RFA for colorectal liver metastasis along with outlining local and systemic immunomodulatory changes as plausible explanations behind observed outcomes, which have been used in combination with check-point inhibitors to reinstate the anti-tumour immune responses [14,15,20,21].

Studies have demonstrated positive immunomodulatory changes following radiofrequency ablation in metastatic liver cancer; however, owing to the involvement of multiple treatments, it would be difficult to exclude the immunological effect of other modalities [22,23]. Herein, we describe the immunomodulatory changes following RFA in advanced metastatic liver cancer secondary to the CRC patient, who refused to receive any adjuvant chemotherapy, which gave a temporal window to study the sole impact of RF on the CLM patient’s immune system.

## 2. Case Presentation

**Figure 1:**
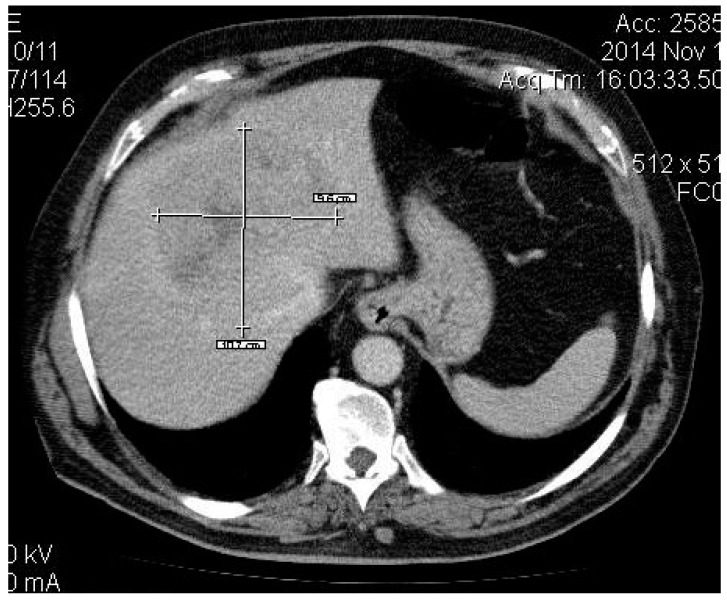
CT revealed a giant metastatic lesion located centrally, mainly in segment 4.

**Figure 2:**
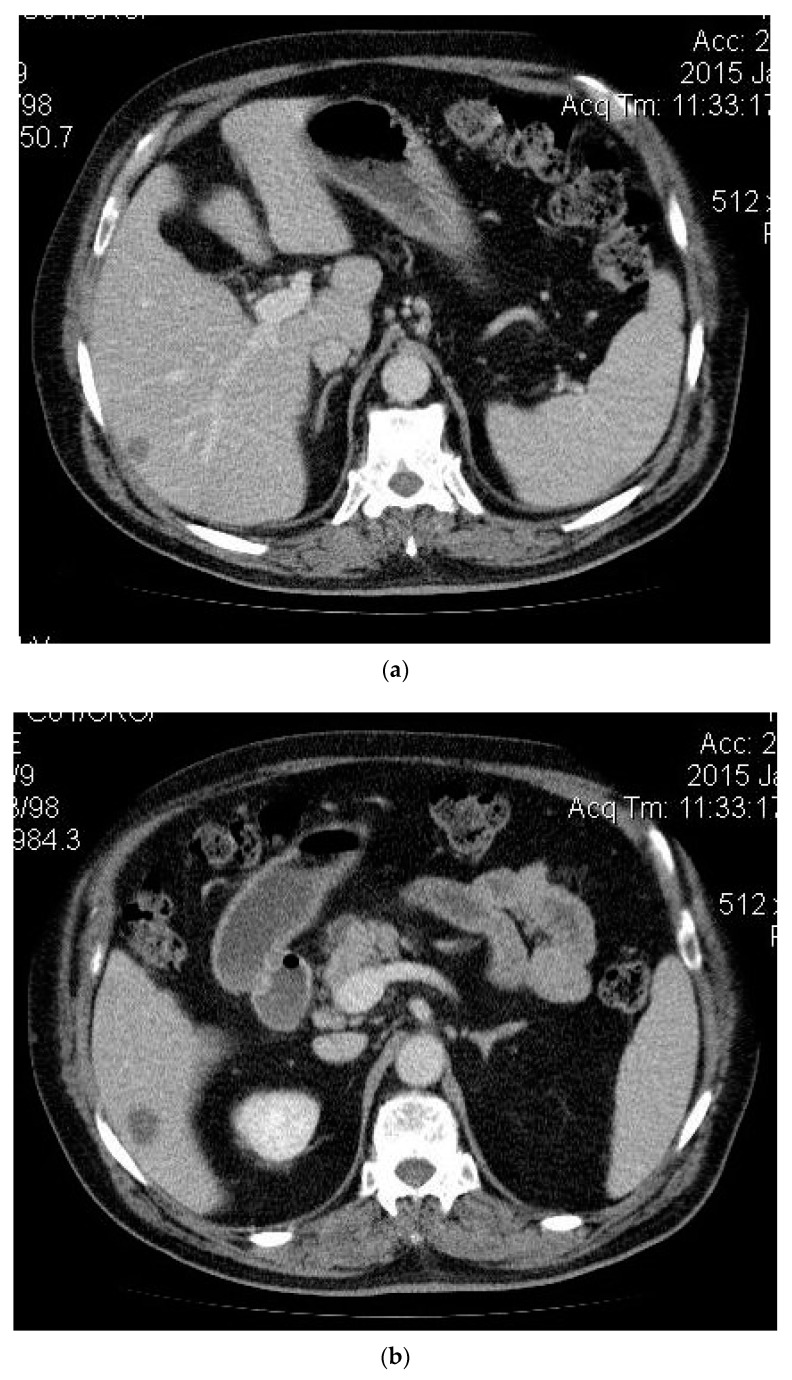
Preprocedure CT. (**a**) Metastatic lesion mass-1; (**b**) Metastatic lesion mass-2.

A 63-year-old man presented in June 2014 to the High Technology Medical Center of Tbilisi (Georgia) with right upper quadrant pain following approval by the Ethic Committee of Tbilisi State Medical University (# 44/3 04.06.2014). On further evaluation, a diagnosis of colonic cancer (sigmoid) was established, and in 2013, he underwent sigmoid bowel resection. The histopathological analysis of the specimen showed a moderately differentiated adenocarcinoma and confirmed tumour-free resection margins. He did not receive any adjuvant treatment. He remained asymptomatic until May 2014, when he developed features of pain in his right hypochondrium and fatigue. Computed tomography (CT) revealed a 10.7 cm lesion in segment 4 and two smaller lesions in segment 6 with imaging characteristic of CLM (see Figure 1, Figure 2a,b), and a diagnosis of TXNXM1, stage IV was made. A biopsy was performed in December 2014, which revealed moderately differentiated adenocarcinoma in the liver, and a diagnosis of liver cancer secondary to CLM was established. He received super-selective TAE using 100–300 μm and 300–500 μm diameter microspheres in December 2014 for the 4 cm lesion in segment 4 of the liver with a complete obliteration of the tumour feeder branches. The post-procedural CT scan revealed a partial technical response while the remaining tumour in the cranial segment remained vascular.

The patient refused any chemotherapy; he underwent a second session of TAE for the same lesion in March 2015. The patient tolerated the embolization procedures well, experiencing just the insignificantly revealed postembolization syndrome lasting for a few days after the procedure. CT was performed 10 weeks after the second TAE session; a complete response was documented showing a necrotic lesion in segment 4 with no evidence of arterial vascularization.

**Figure 3:**
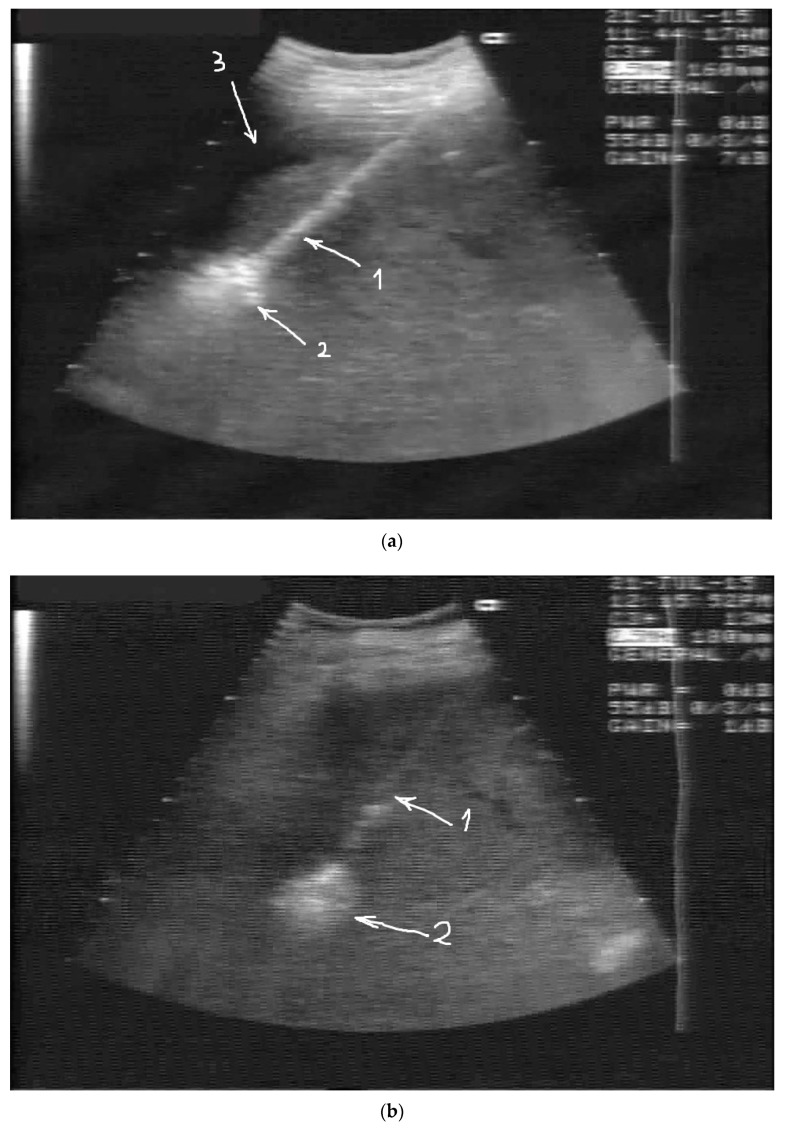
(**a**) US-guided RFA ablation of mass 1. 1—RFA electrode, 2—hyperechoic cloud showing the ablation process, 3—fluid in the peritoneal cavity, injected for hydrodissection. (**b**) US-guided RFA ablation of mass 2. 1—RFA electrode, 2—hyperechoic cloud showing the ablation process.

**Figure 4:**
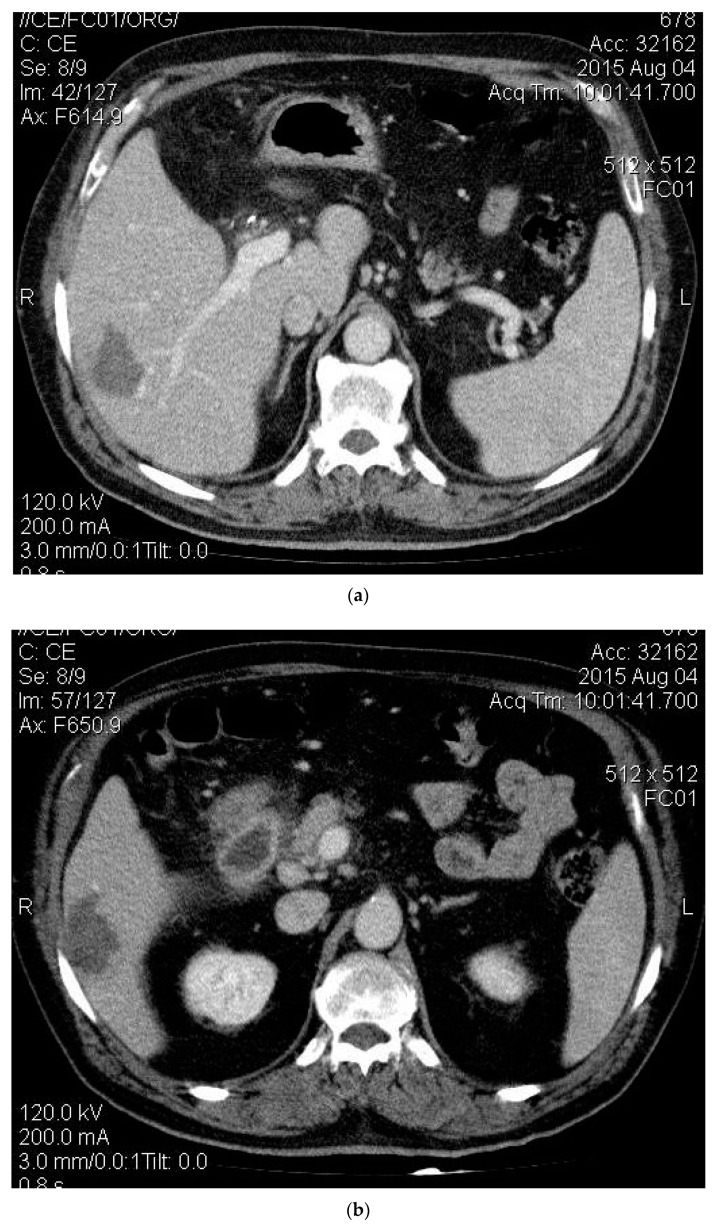
(**a**) The complete ablation of mass 1; (**b**) The complete ablation of mass 2.

**Figure 5:**
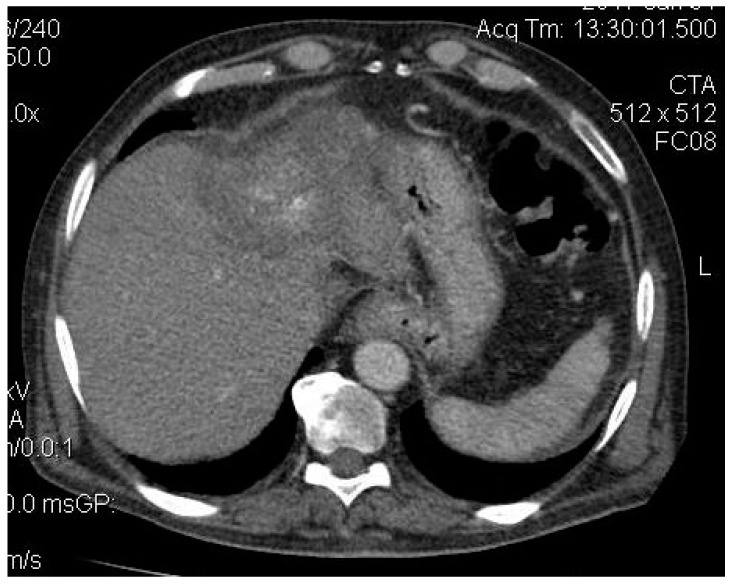
Lesion response to the embolization—tumour is calcified and shrunk.

The right lobe’s two smaller masses grew from 14 and 21 mm to 18 and 29 mm, correspondingly, as documented by CT 3 months after the second TAE session, and were treated by percutaneous RFA. Due to the subcapsular location of the lesions, hydrodissection was performed through percutaneously positioned 8 Fr diameter drainage catheters, and around 3000 mL of glucose solution was injected into the peritoneal cavity to achieve 10 mm of separation between the liver surface with the abdominal wall and the diaphragm (Figure 3a,b). No complications occurred during the procedure. A repeated CT scan was performed one month after RFA ablation, demonstrating the complete response to it (Figure 4a,b). The segment 4 lesion appeared to be necrotic and reduced in size, but there was evidence of this mass’ revascularization from the gastrohepatic trunk, which was not seen in the previous CT scan (Figure 5). A third session of TAE in November 2015 for the segment 4 lesion was performed because of this, while the prior ablated masses did not show any features suggestive of recurrence. After this, the patient received a short course of nexatinplus- and capecitabine-based chemotherapy.

The follow-up CT scan performed six months later showed multiple new satellite lesions in both lobes. The decision was made to start oxaliplatin-based chemotherapy in combination with capecitabine (Xeloda) in April 2017, and a follow-up CT did not show any changes.

Further evaluation in October 2017 showed a progression of the disease on CT; chemotherapy started with 350 mg irinotecan infusions, which the patient received until 15 March 2018. In spite of this, he did not show any improvement; liver failure developed later, and he died two years and nine months after the RFA procedure. Concurrently, we explored the impact of RFA on the adaptive immune response in the absence of the influence of any other therapeutic modality, as he refused any other form of treatment. Peripheral blood samples were obtained before and after one and three months of RFA. The immunophenotypic analysis was accomplished within 24 h of sample collections for a panel of cellular and cytokine subsets including CD39^+^CD4^+^, CD4^+^ T cells, IL-10, IL-17, INF-γ and TGF-β. Additionally, the neutrophil/lymphocyte ratio (NLR) and platelet/lymphocyte ratio (PLR) were calculated as parameters of systemic inflammation.

**Figure 6:**
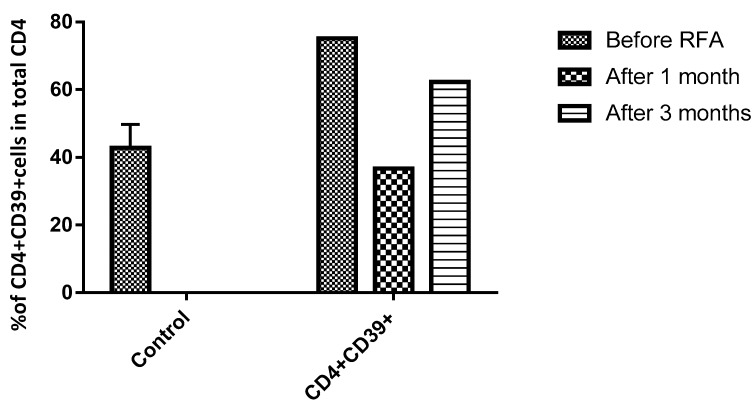
The frequency of CD39^+^CD4^+^ T cells in the peripheral blood. The percentage of CD39^+^CD4^+^ cells in total CD4^+^ T cells was decreased after 1 month and increased after 3 months, but did not reach the index before the RFA procedure.

In contrast to the percentage of CD39^+^CD4^+^ cells to the total CD4^+^ T cells in normal subjects, which is approximately 10%, the preprocedural (RFA) value was 75.2%; however, following one month of RFA, it declined to 36.7 % but rose again after three months to 62.3% (Figure 6).

**Figure 7:**
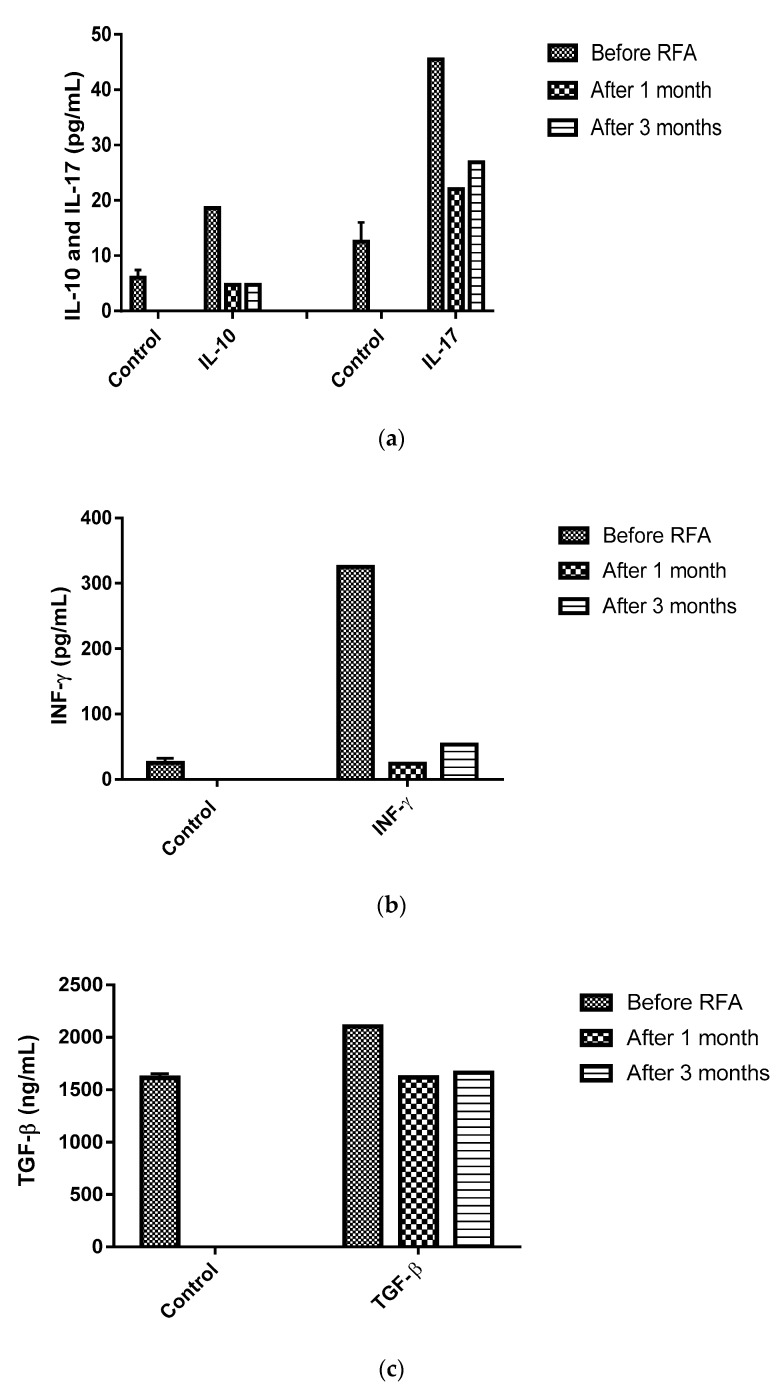
Altered levels of serum cytokines in the patient with colorectal liver metastasis before the RFA procedure and one and three months after the procedure. The concentrations of (**a**) IL-10 (upper panel) and (**b**) INF-γ (lower panel) decreased after one month and were not modified three months after the RFA procedure. The concentration of IL-17 (a) decreased after one month and slightly increased after three months. (**c**) The concentration of TGF-β decreased after one month and was not modified three months after the RFA procedure.

Similarly, we observed elevated levels of IL-10, TGF-β, IFN-γ and IL-17, which declined one month following RFA and remained unchanged until three months (see Figure 7a–c).

These changes in cytokine levels as well as the CD39^+^CD4^+^ T cells’ frequency were accompanied by a reduction of gamma-glutamyl transferase (GGT) and systemic inflammatory markers NLR and PLR, while ALT and AST increased at one month and decreased at three months after RFA (Table 1).

However, further assessments of immunomodulatory changes were not made, as the patient decided to opt for chemotherapy.

## 3. Discussion

Recent reports have indicated that thermal ablation can potentially activate host antitumor immunity and may be of benefit in metastatic control and the suppression of long-term tumour resistance [24,25,26]. Fagnoni et al. [27] reported that radiofrequency ablation of primary and metastatic liver tumours was effective in boosting anti-tumour immune response through the induction of heat shock proteins on tumour cells and had an acute phase response which caused the release of pro-inflammatory cytokines and the mobilization of antigen-presenting cells and effector lymphocytes. Studies have demonstrated that various inflammation-based factors, including the PLR and NLR, have prognostic value in the management of cancer patients [28]. Platelets can promote tumour growth by increasing angiogenesis via the cytokine vascular endothelial growth factor (VEGF) [29]. Wiesner et al. [30] demonstrated that the platelet content of VEGF-A in cancer patients was significantly increased compared to healthy controls. Kwon et al. [31] outlined that patients with greater PLR had an increased likelihood of a positive lymph node ratio in CRC.

Systemic inflammatory markers such as NLR are useful prognostic markers for various types of cancers [32,33,34]. Pre- and/or post-treatment NLR and/or PLR are predictive of clinical outcomes in patients receiving selective internal radiation therapy. The survival of patients with increased NLR was shown to be significantly poorer than that the survival of patients with decreased NLR [35]. Consistent with previous reports, the present case demonstrated that the RFA treatment decreased the NLR and PLR ratio in the peripheral blood of the patient (Table 1).

Cytokines and cells of tumour microenvironments establish a suppressive milieu in growing cancers. Various studies have demonstrated that CD39 was implicated in promoting tumour growth and metastases through the suppression of antitumor immune responses and enhancement of angiogenesis [36]. Ectonucleotidase CD39 expression by regulatory T lymphocytes and the associated ATP dephosphorylation is one of the poignant mechanisms responsible for the suppressive activity of these cells and for the growth of metastatic tumours in the liver. Our clinical case shows that RFA might stimulate antitumor immunity through the inhibition of CD39^+^ on circulating CD4^+^ T cells, which is an indicator of a good prognosis (Figure 6).

Okumoto et al. [37] studied the relationship of plasma TGF-β to antitumor immunity and found higher levels of TGF-β in liver cancer patients versus healthy individuals, which might be associated with a malignant transformation or progression of chronic disease likewise to IL-10. Additional studies demonstrated similar associations between a high infiltration of IL-17-producing cells in the peritumoural stroma and the progression of liver cancer via promoting angiogenesis and expressing macrophages stimulated by inflammatory cytokines (IL-1β, IL-10 and TNF-α by IL-17) [38,39]. Nevertheless, the data from experimental studies exploring the involvement of Th17 in tumour immune surveillance are still inconclusive [40]. We believe that the beneficial effect of RFA on anti-tumour response is achieved through the downregulation of CD39 expression in the circulating CD4^+^ T cell population and the reduction of the serum levels of anti-inflammatory cytokines IL-10, TGF-β and IL-17, which positively correlate with the diminished GGT levels and the subdued inflammatory markers NLR and PLR. The present case highlights the importance of RF-induced immunomodulatory changes in the improvement of survival rates in advanced colorectal liver metastasis. To the best of our knowledge, this is the first case report where the immunomodulatory effects of RF in colorectal liver metastasis were assessed in the absence of other therapeutic modalities. However, we intend to conduct further investigations to better understand the changes in the levels of cytokines and cellular subtypes, and to discern whether repeated sessions of the RFA procedure trigger a “booster” effect for establishing anti-tumour immunity.

## Figures and Tables

**Table 1:** Blood test results. Blood tests revealed moderate changes of liver enzymes. Neutrophil/lymphocyte ratio (NLR) and platelet/lymphocyte ratio (PLR) levels were decreased during the RFA treatment. RFA: radiofrequency ablation.

Test	Results			Normal Range
	RFA Procedure (in July 2015)			
	Before Procedure	After 1 Month	After 3 Months	
	**Liver Function Tests**			
**ALT**	39.2	59.7	42.9	(≤41 U/L)
**AST**	46.2	64	57.5	(≤40 U/L)
**GGT**	208	118	89	(10–70 U/L)
**Neutrophil/Lymphocyte Ratio and Platelet/Lymphocyte Ratio**				
**NLR**	10.3	5.3	5.91	2.4
**PLR**	202	203	156	100–150
